# Supplementary material for: Discovering Potential Compounds for Venous Disease Treatment through Virtual Screening and Network Pharmacology Approach
Source: Molecules. 2023 Dec 5;28(24):7937. doi: 10.3390/molecules28247937 (PMC10745828; doi:10.3390/molecules28247937)

Supplementary figures

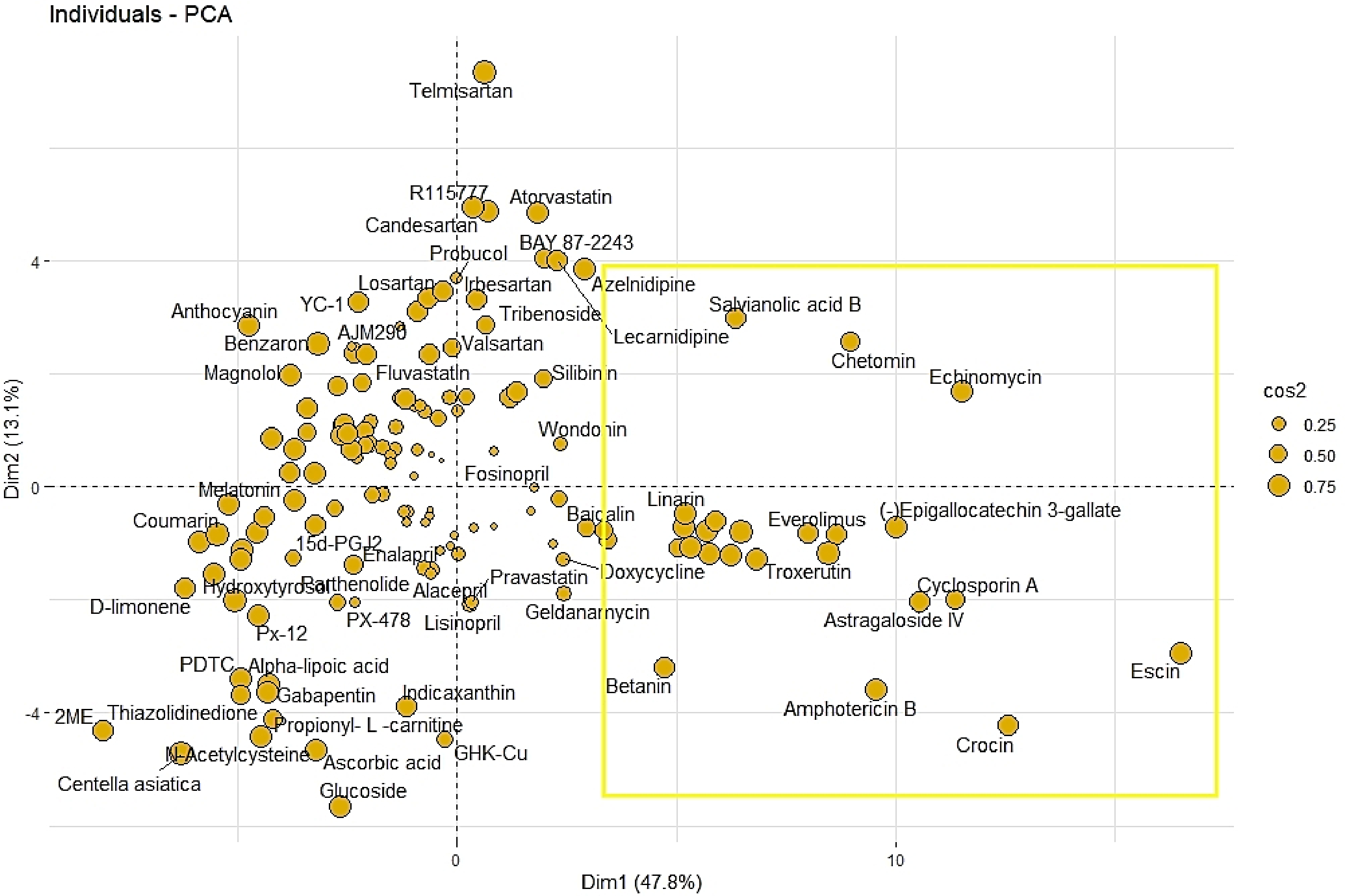

Figure S1. Principal component analysis(PCA) scoreplot of the analyzed reference compounds useful in VD with respect to their molecular descriptors: GPCR.ligand, Ion.channel.modulator, Kinase.inhibitor, Nuclear.receptor.ligand, Protease.inhibitor, Enzyme.inhibitor, nviolations, natoms, log.Kp..cm.s., Lipinski..violations, Ghose..violations, Veber..violations, Egan..violations, Muegge..violations, Bioavailability.Score, Molweight, cLogP, cLogS, H.Acceptors, H.Donors, Total.Surface.Area, Polar.Surface.Area, Druglikeness, Shape.Index, Molecular.Flexibility, Electronegative.Atoms, Rotatable.Bonds, Aromatic.Rings, Aromatic.Atoms, sp3.Atoms, and Symmetric.atoms.

Supplementary data S2 **Escin clustering**

A

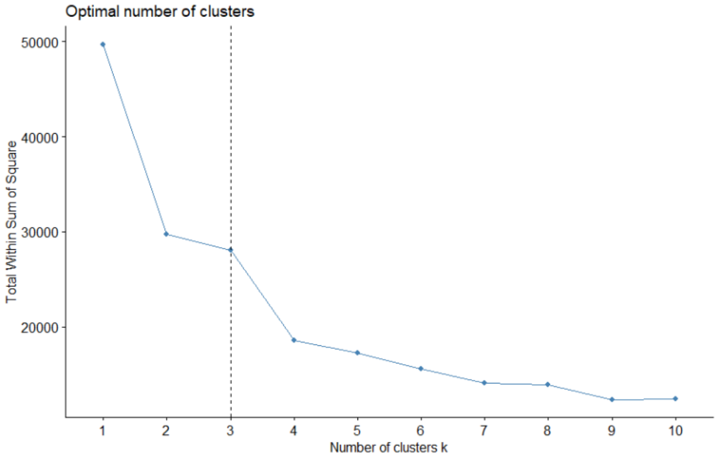

B

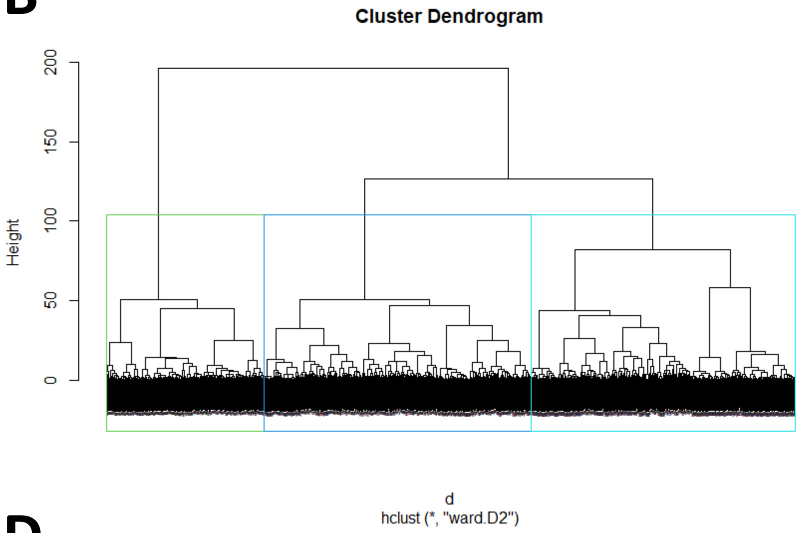

C

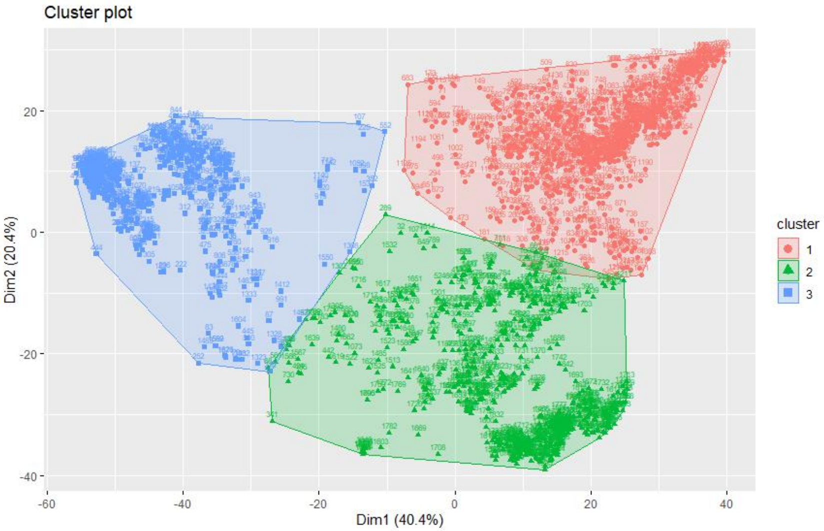

D

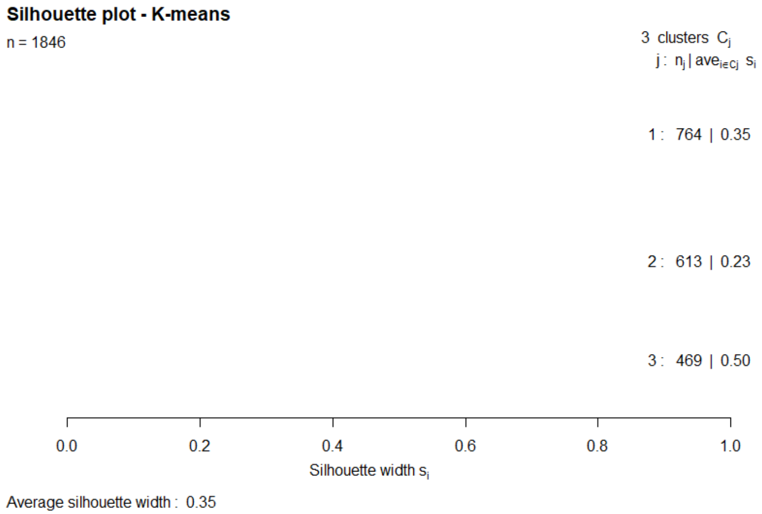

Supplementary data S2 **Crocin clustering**

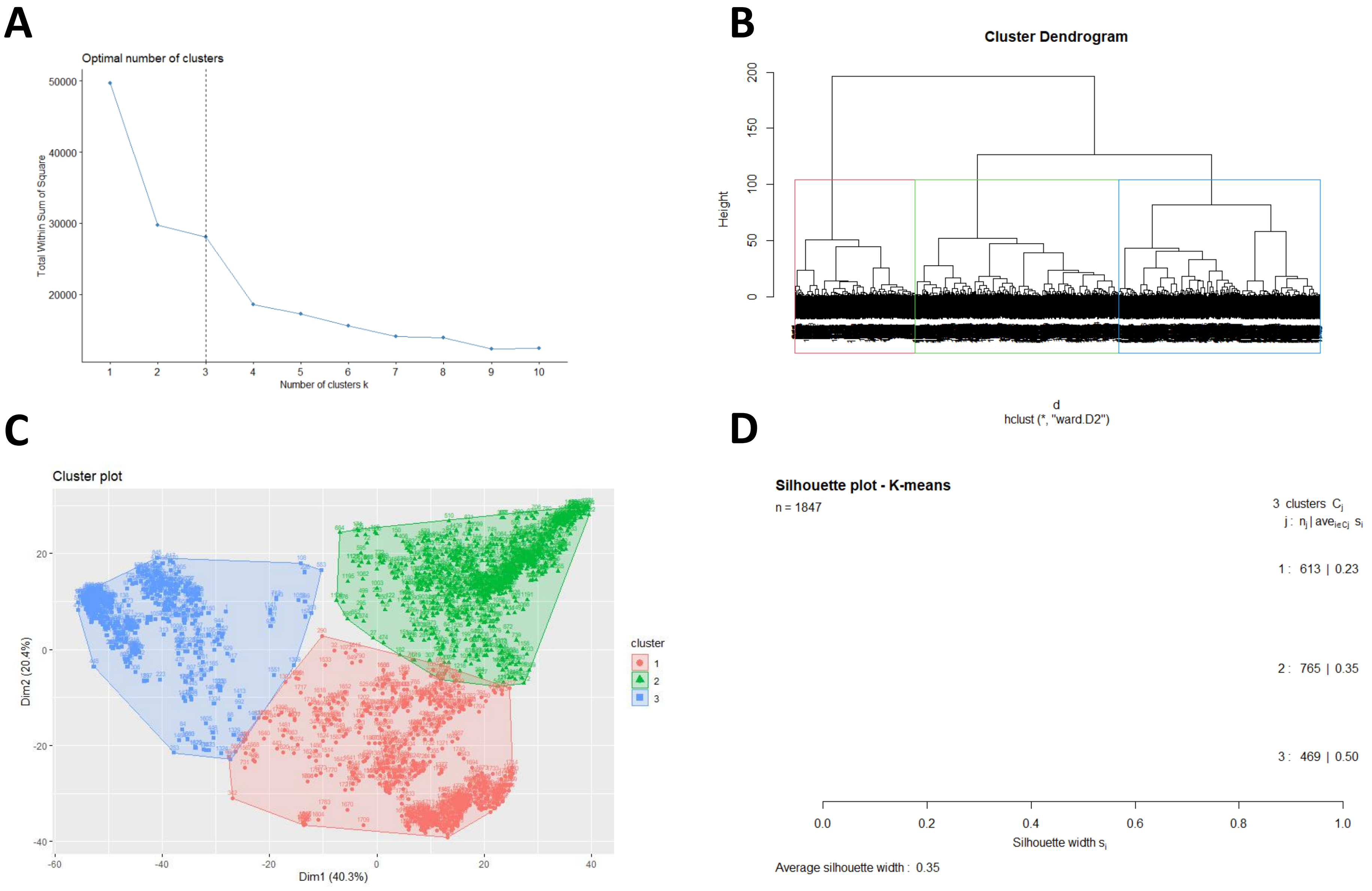

Supplementary data S2

Echinomycin clustering

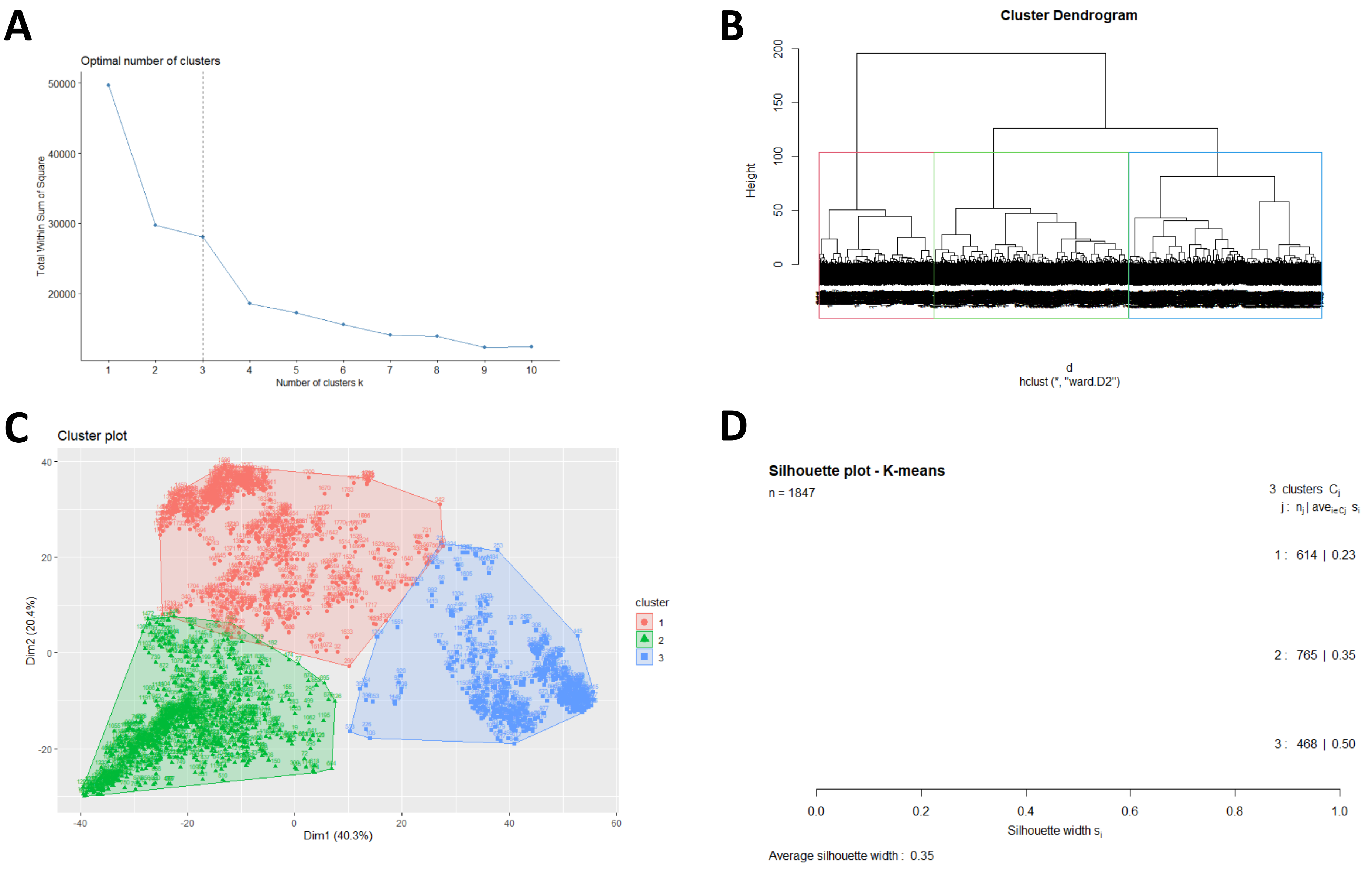

Supplementary data S2

Cyclosporin A clustering

A

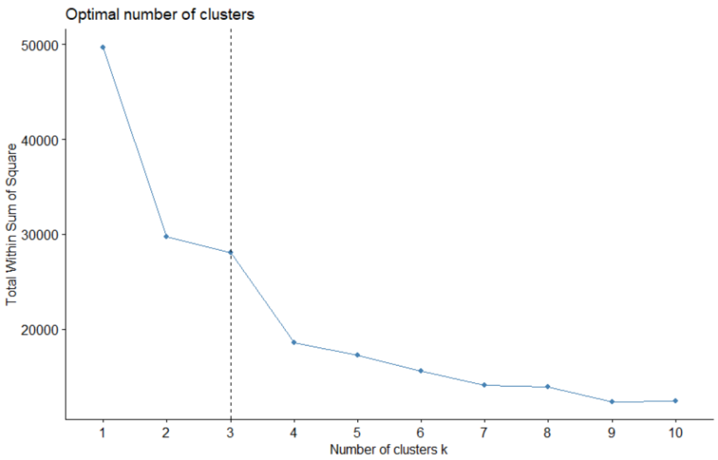

B

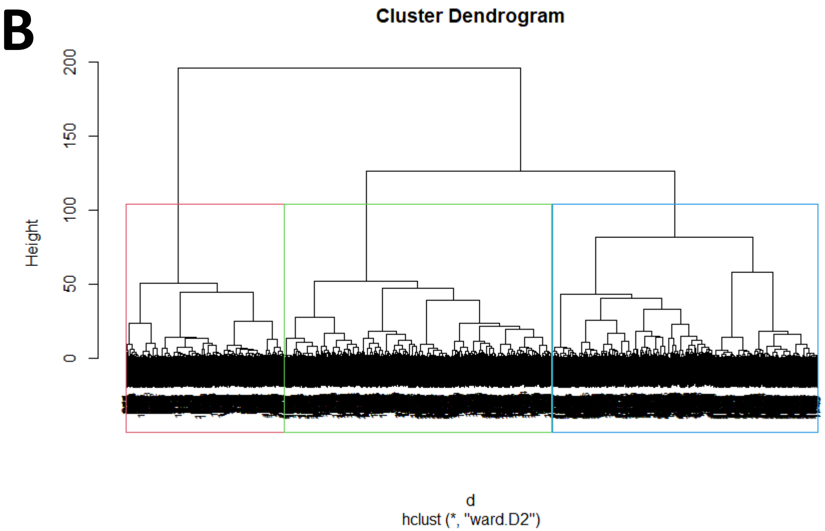

C

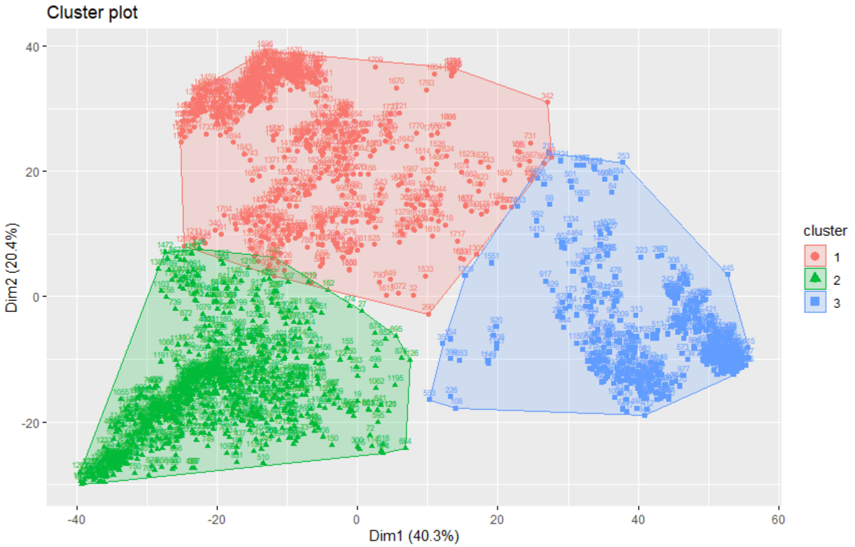

D

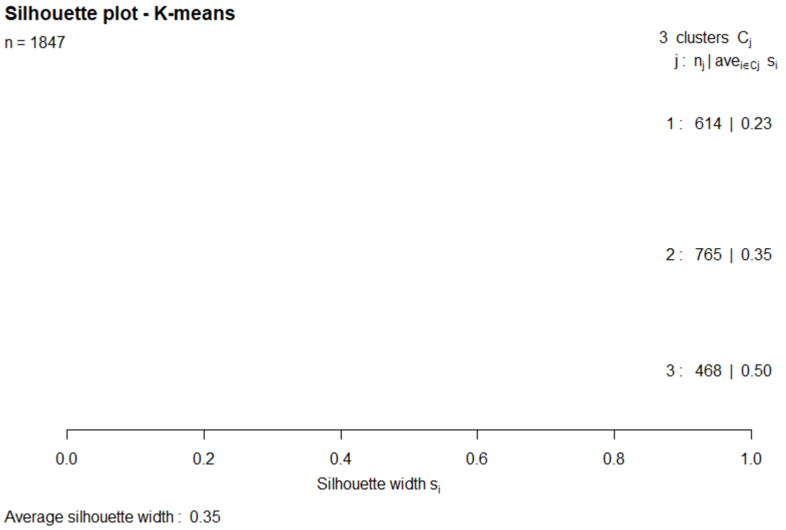

Supplementary data S2

Amphotericin B clustering

A

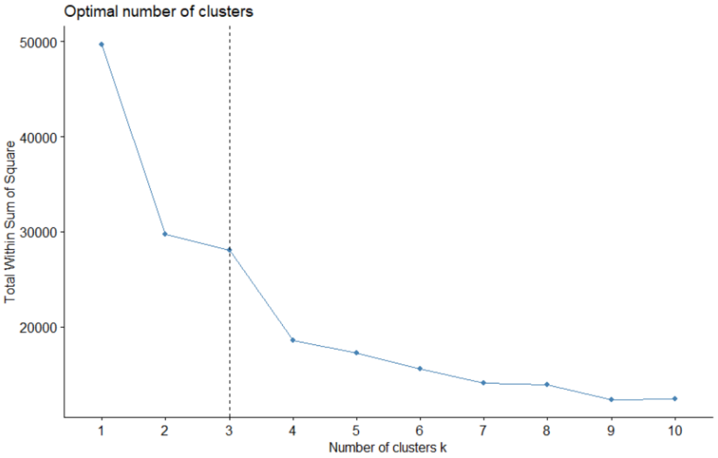

B

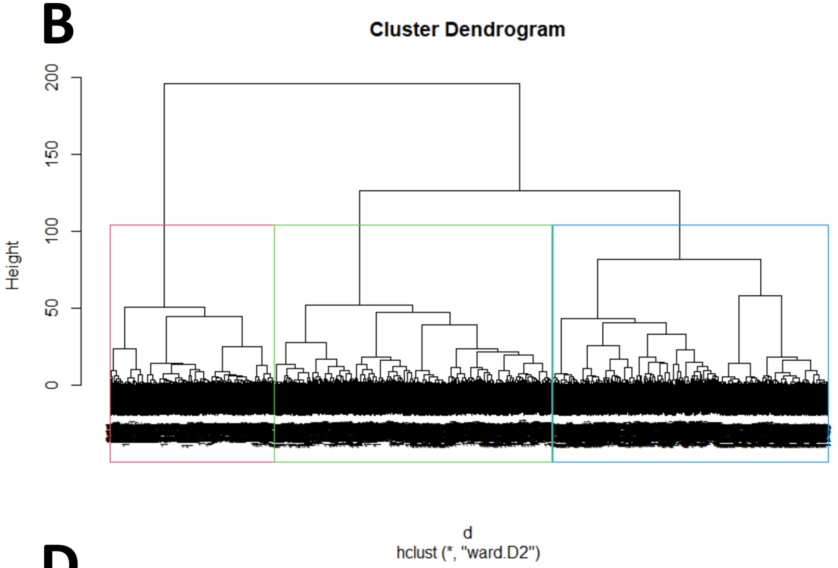

C

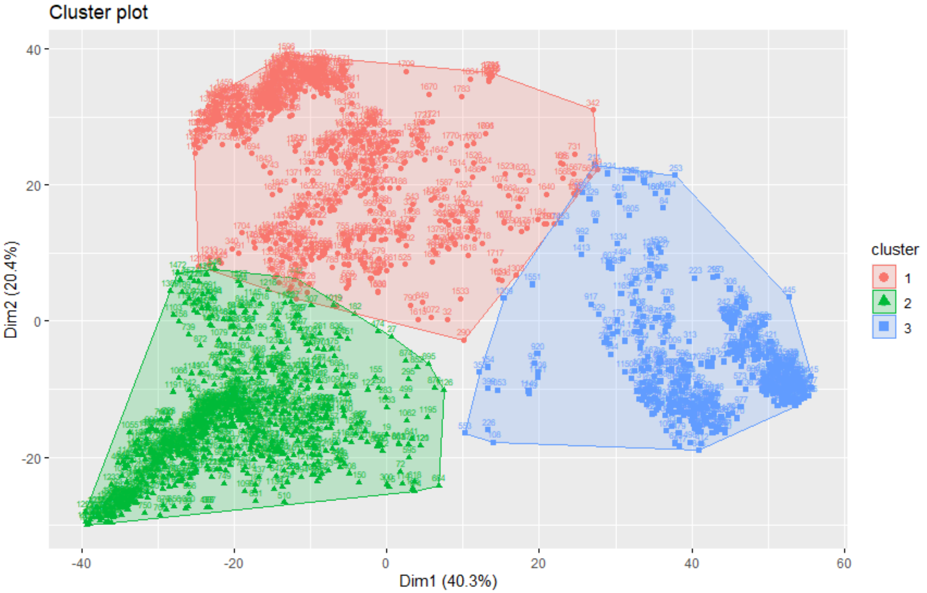

D

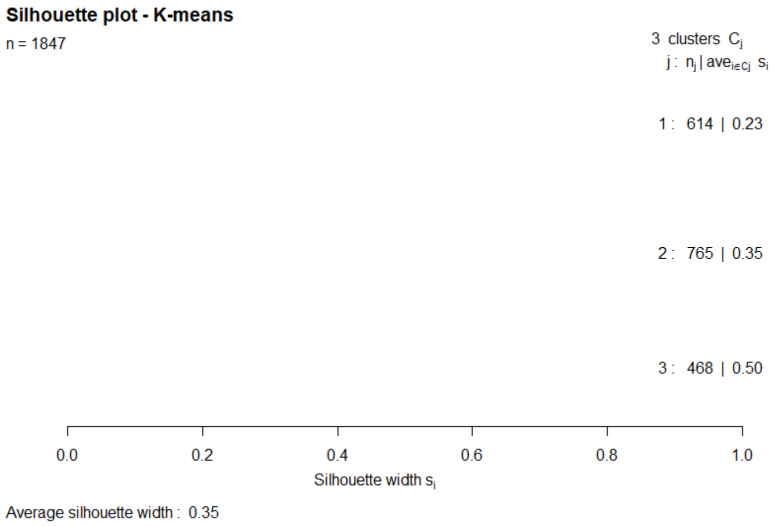

Supplementary data S2

Everolimus clustering

A

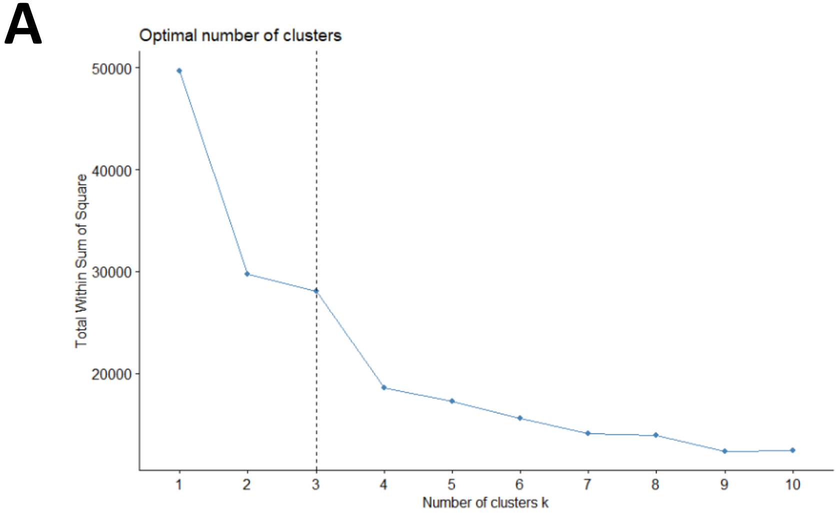

B

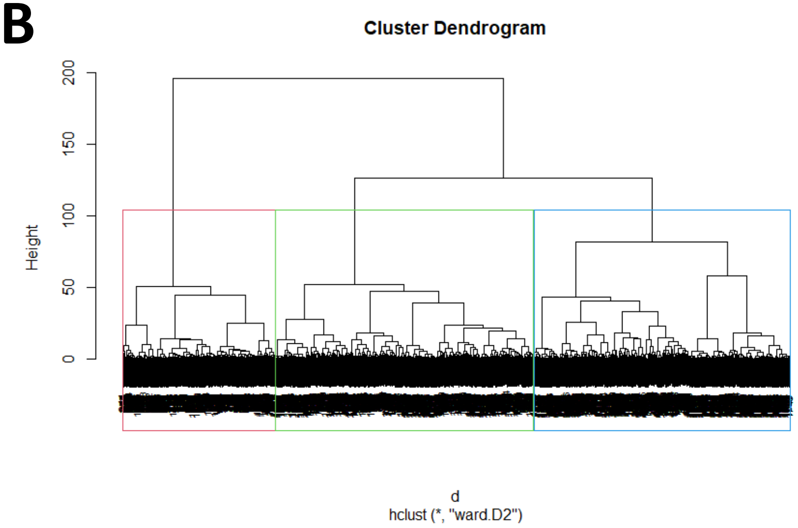

C

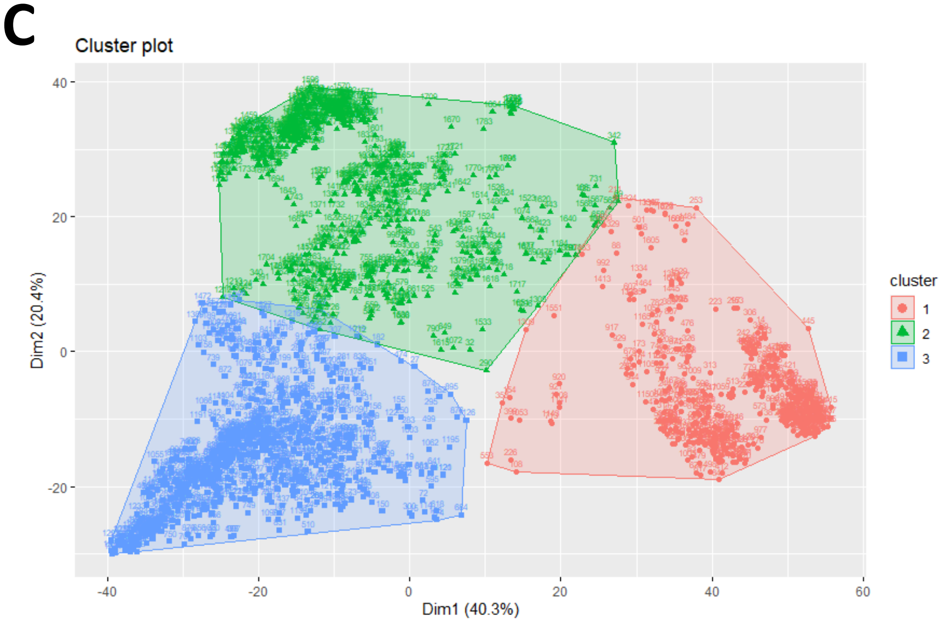

D

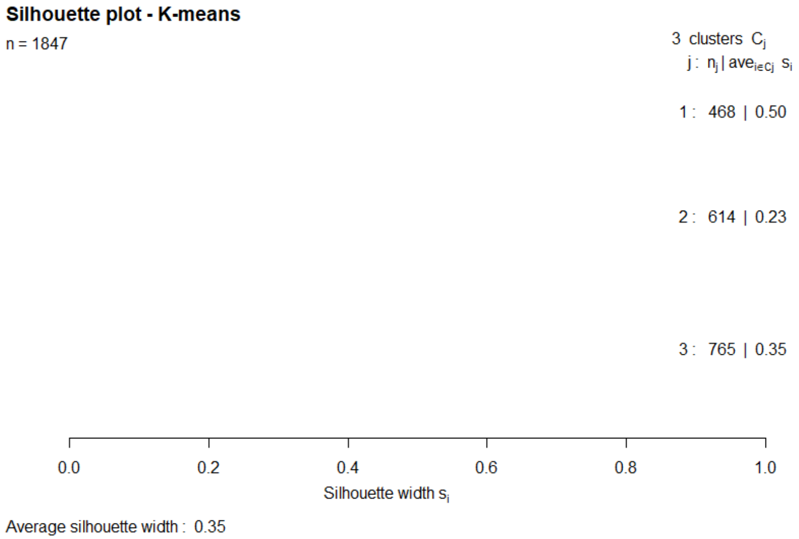

Supplementary data S2

Rapamycin clustering

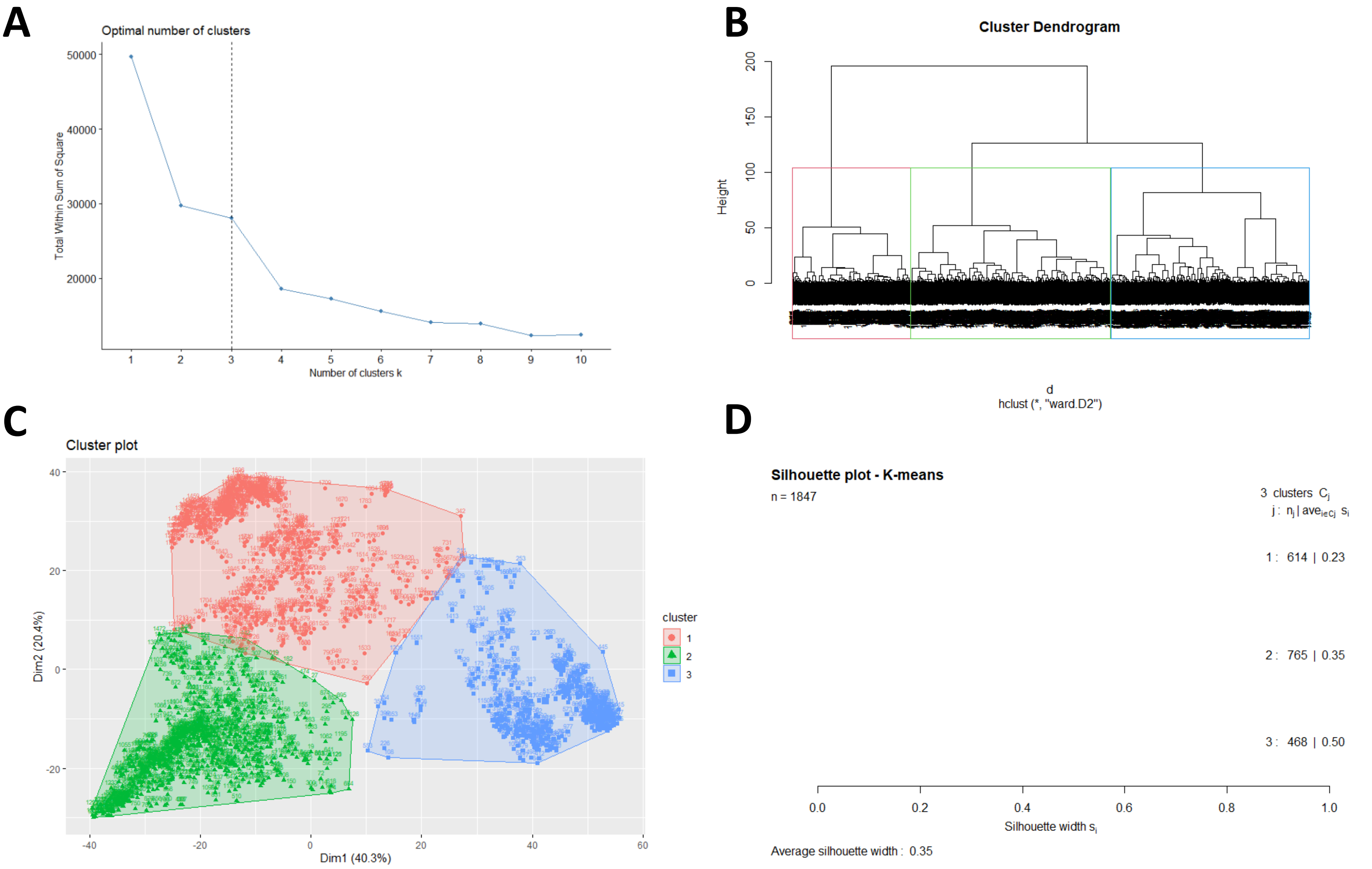

Supplementary data S2

Chetomin clustering

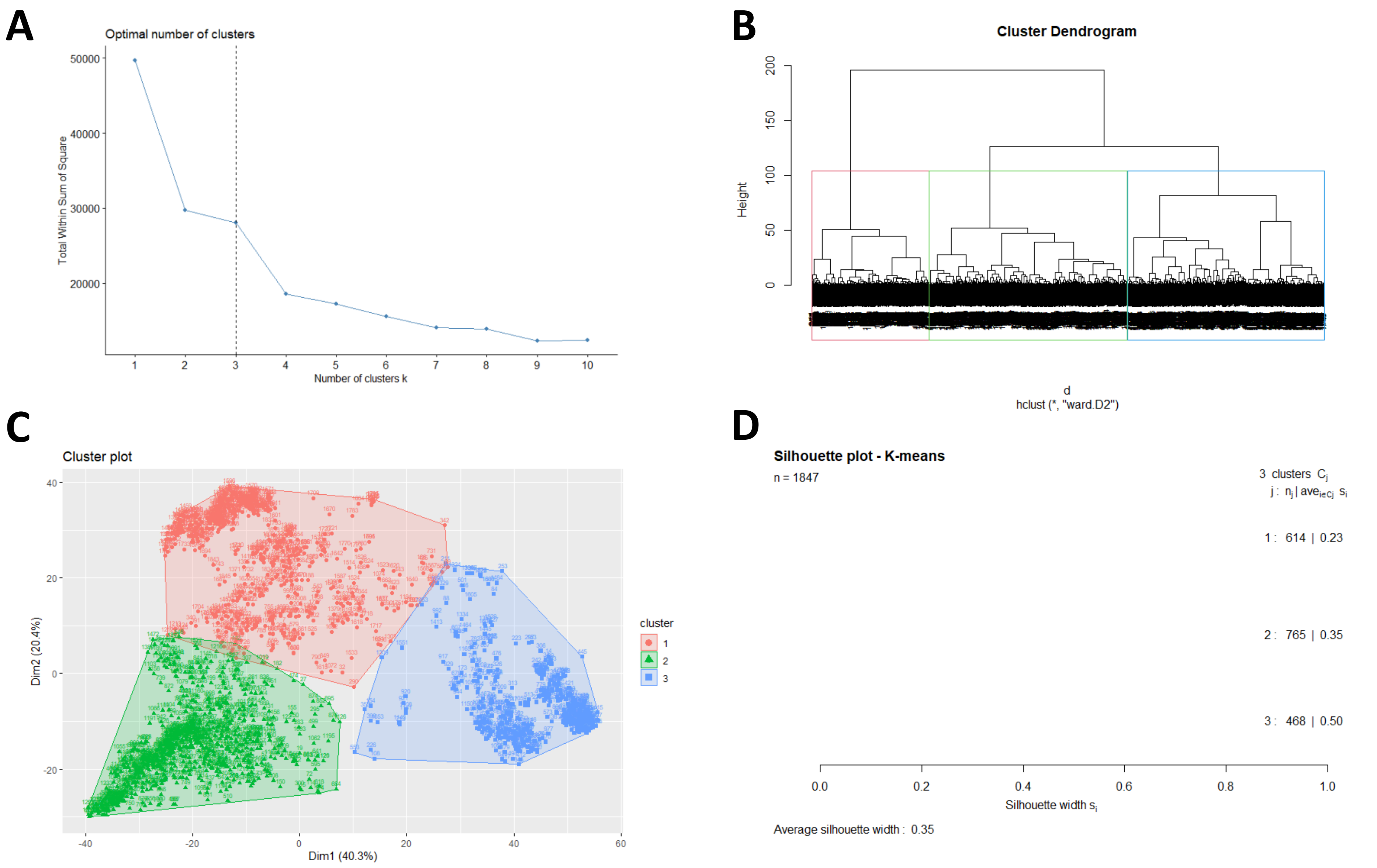

Supplementary data S2

Astragaloside IV clustering

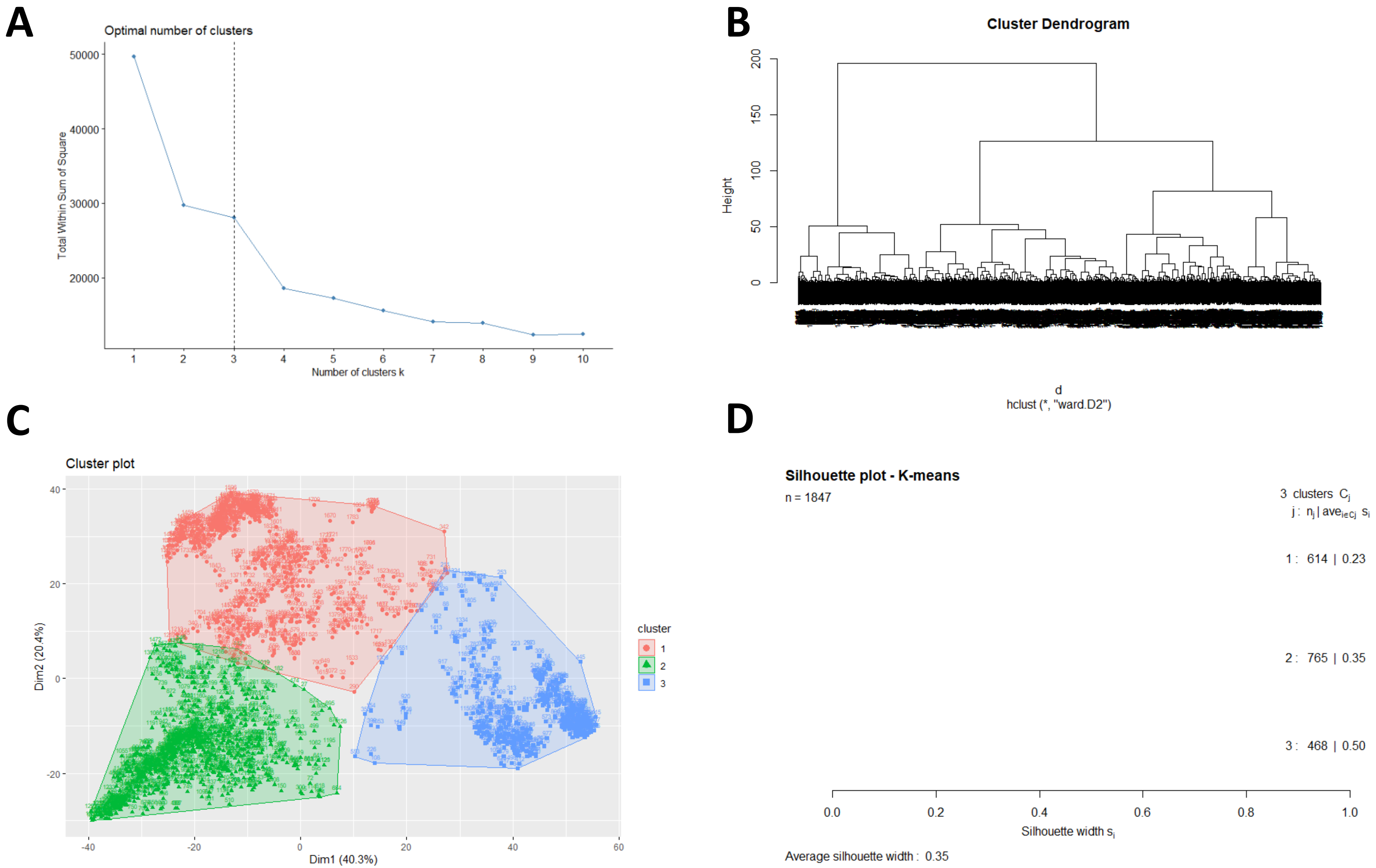

Supplementary data S2

20(R)-ginsenoside Rh2 clustering

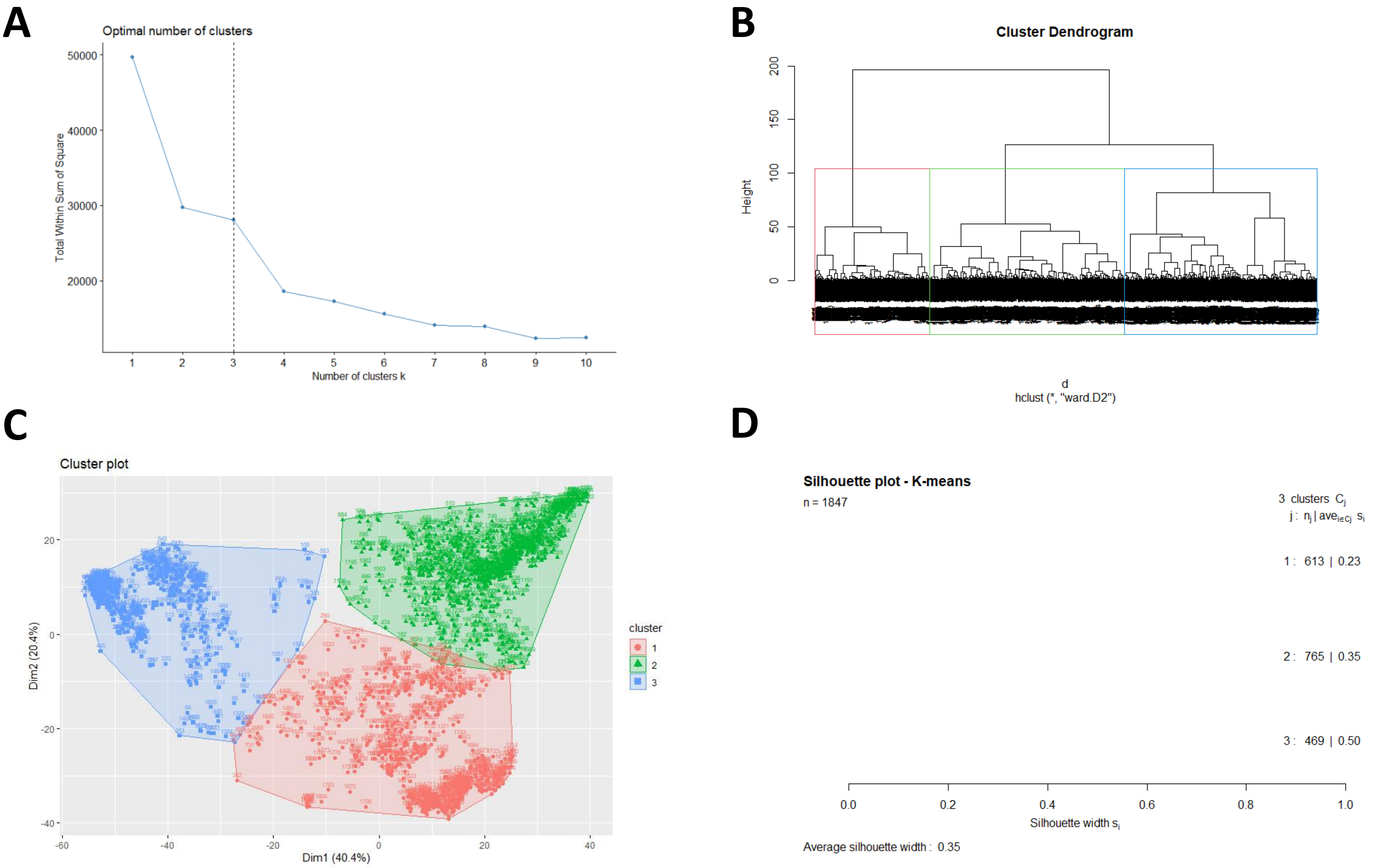

Supplementary data S2 Epigallocatechin 3-gallate clustering

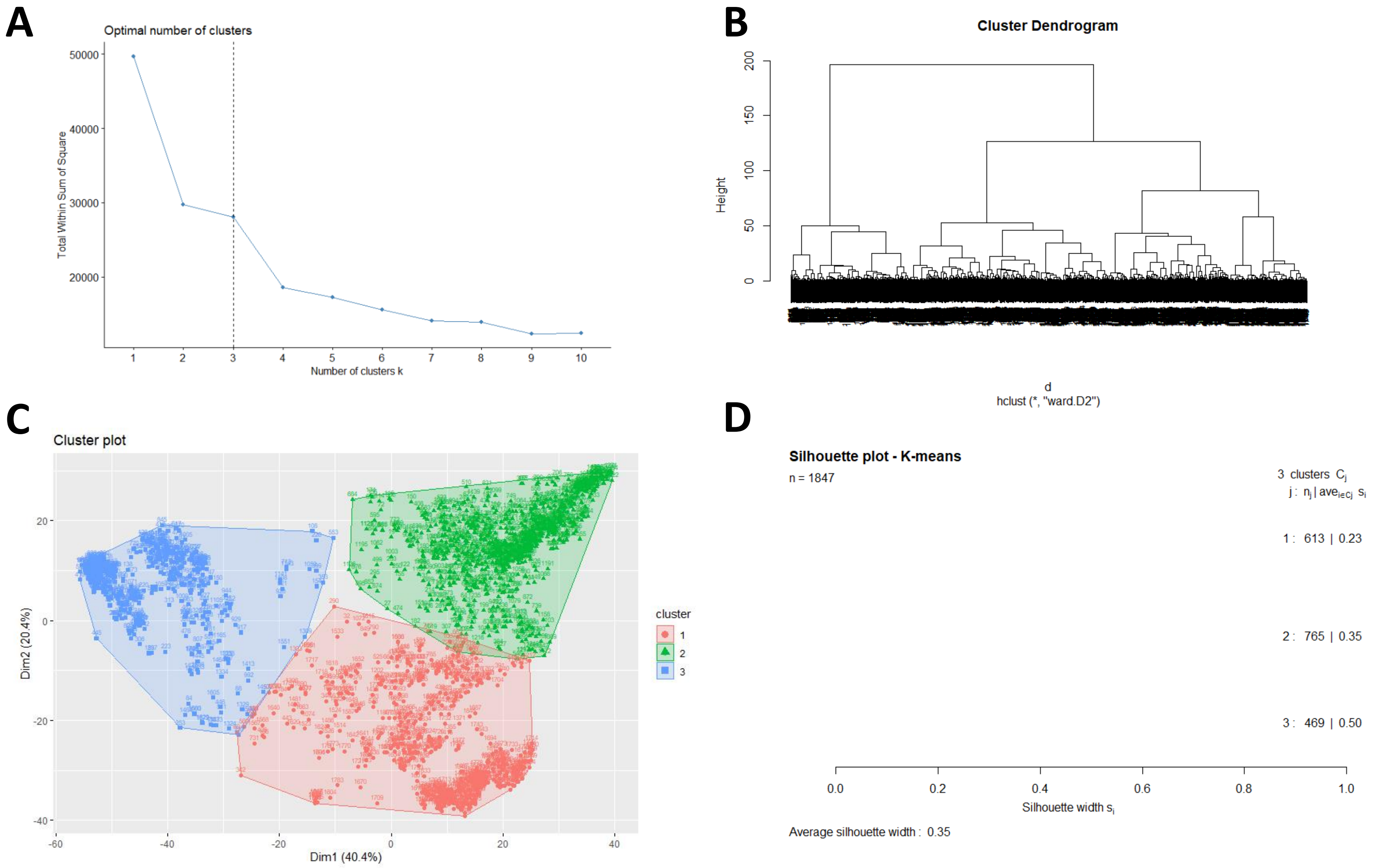

Supplementary data S2

Troxerutin clustering

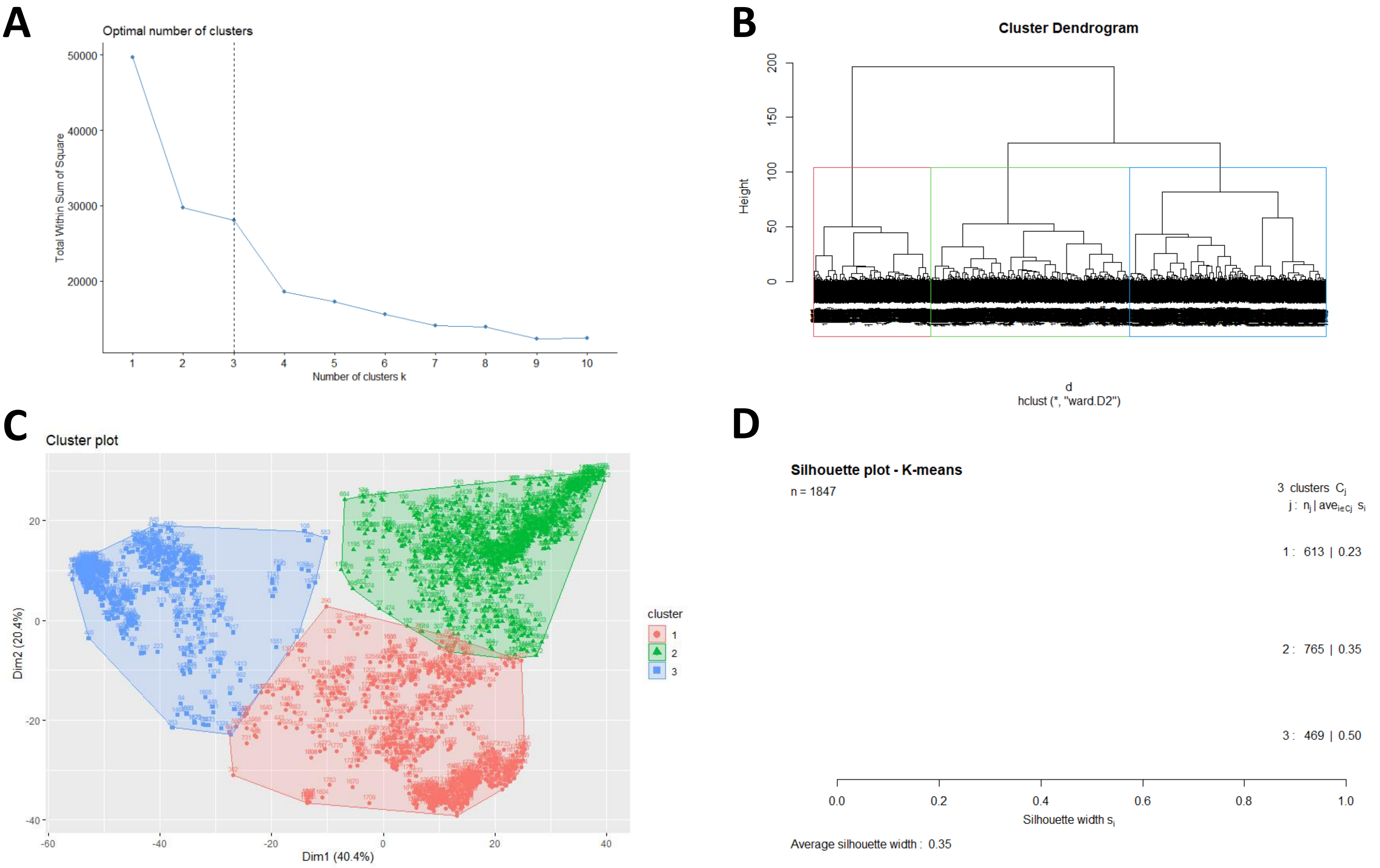

Supplementary data S2 O-(beta-hydroxyethyl)-rutoside clustering

A

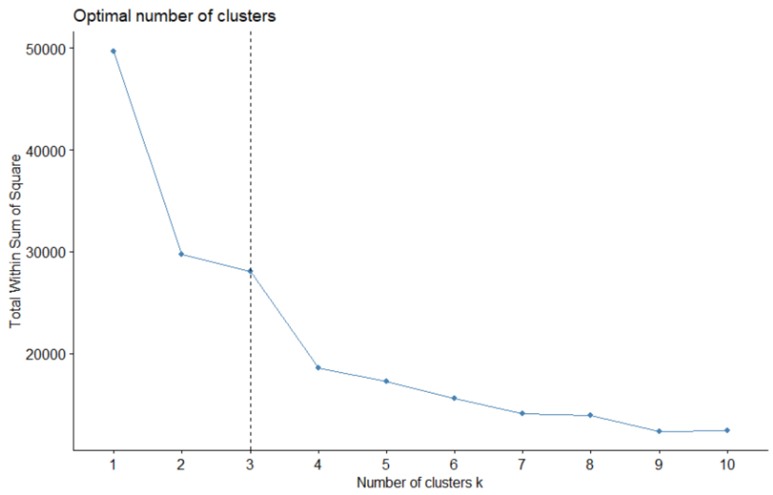

B

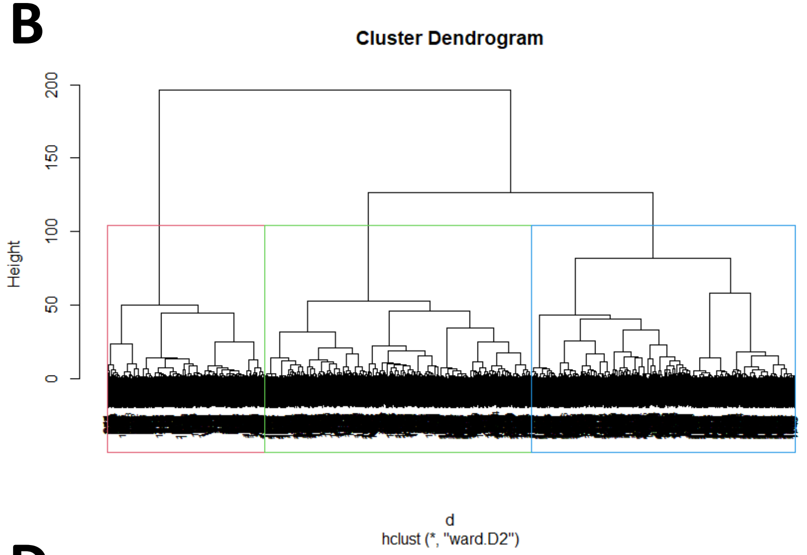

C

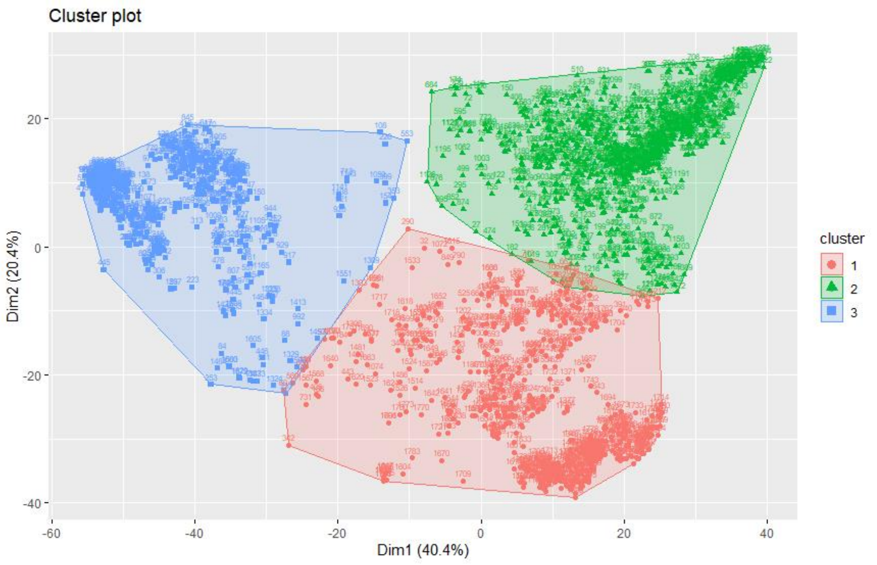

D

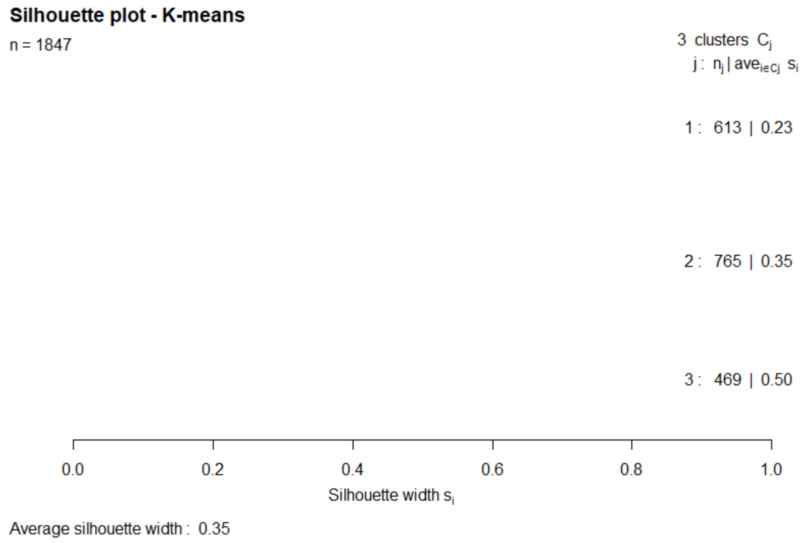

Supplementary data S2

Salvianolic acid B clustering

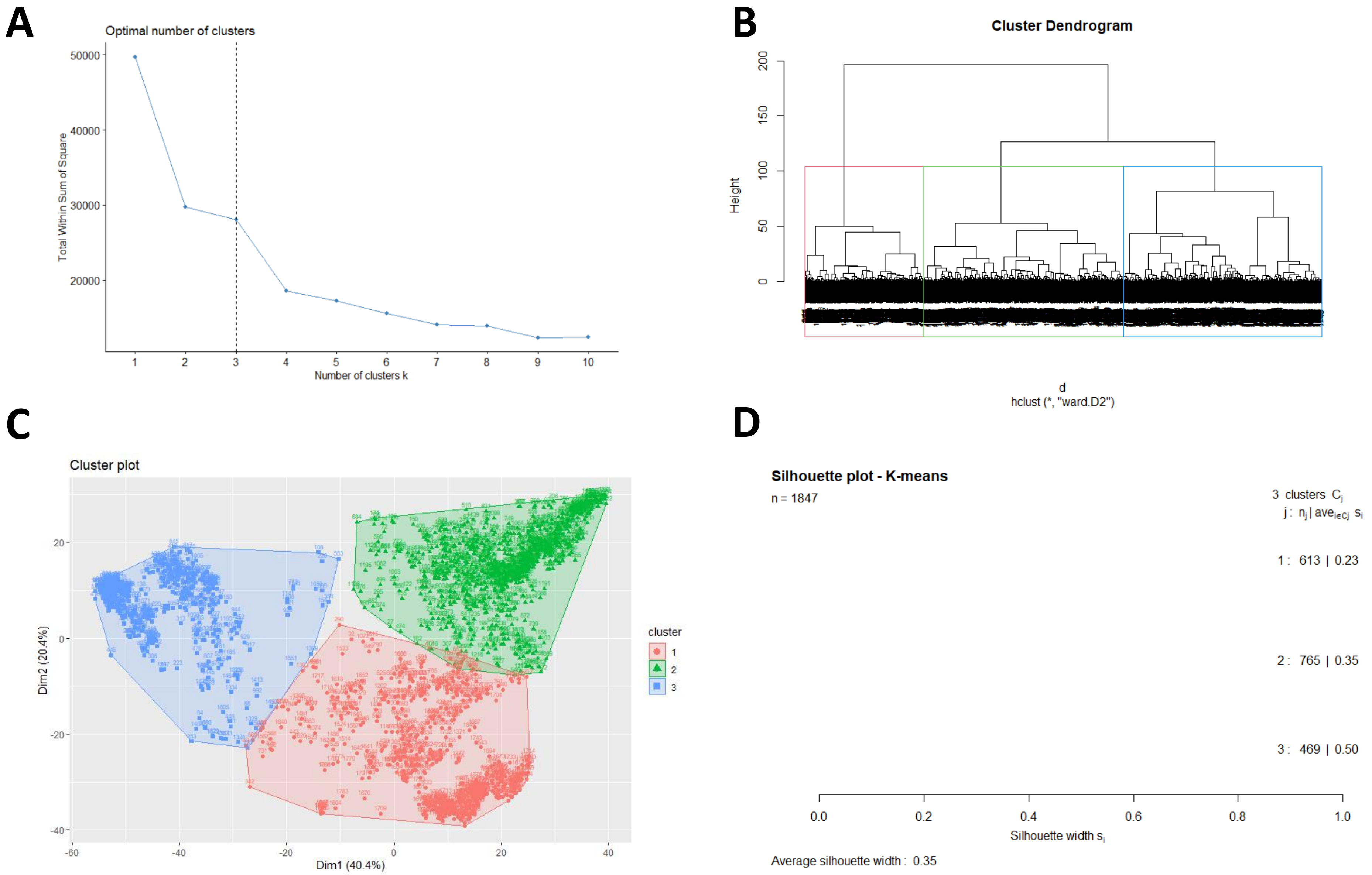

Supplementary data S2

Keracyanin chloride clustering

A

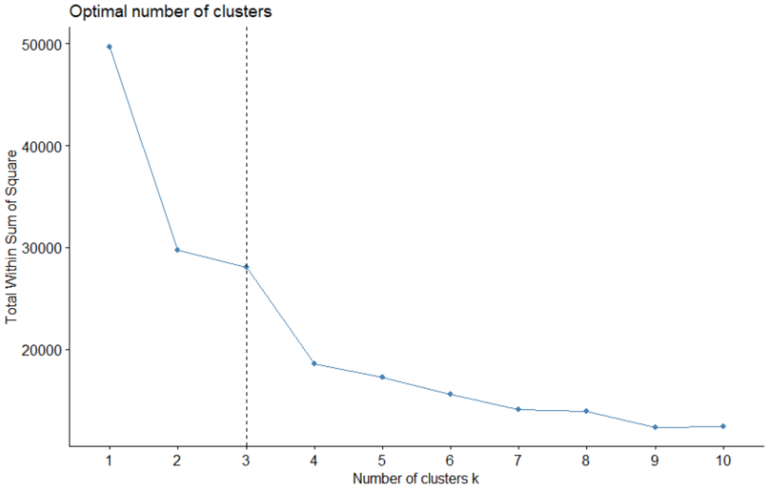

B

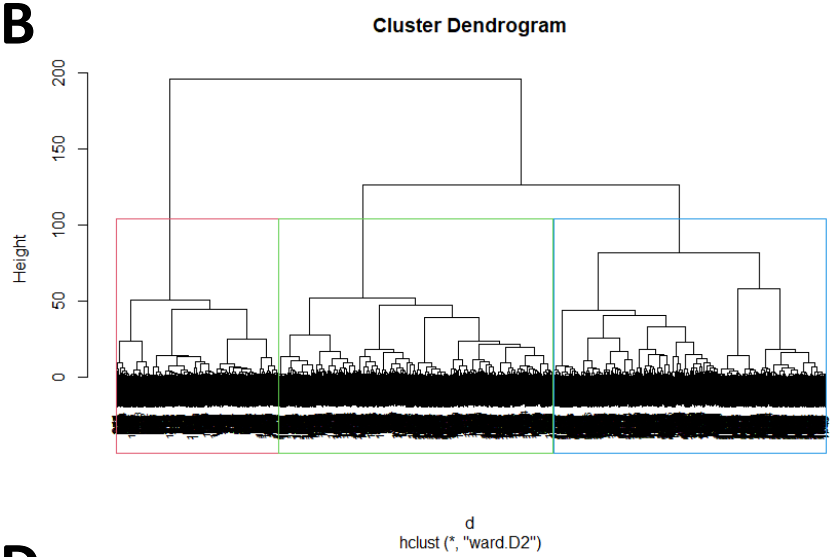

C

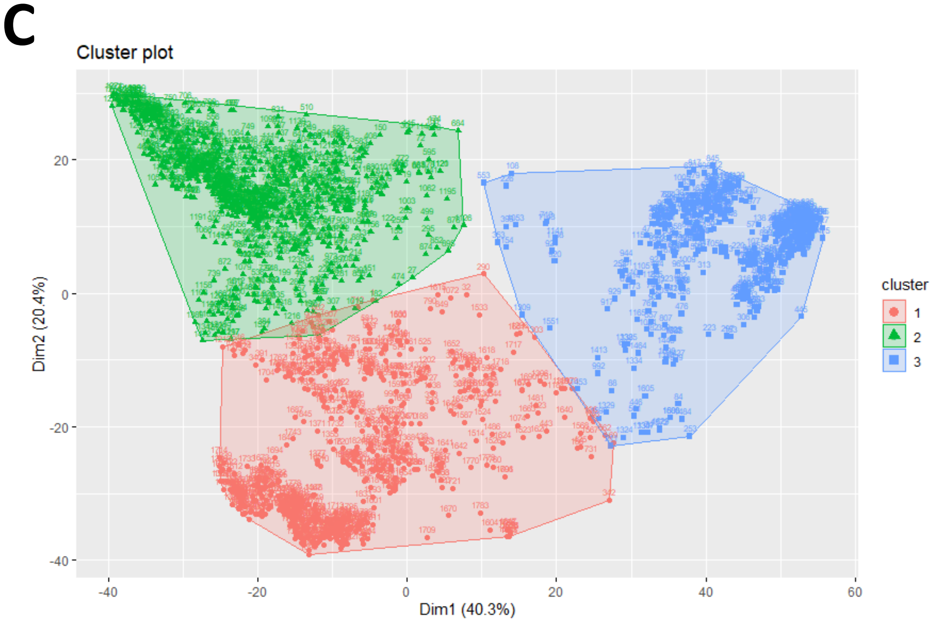

D

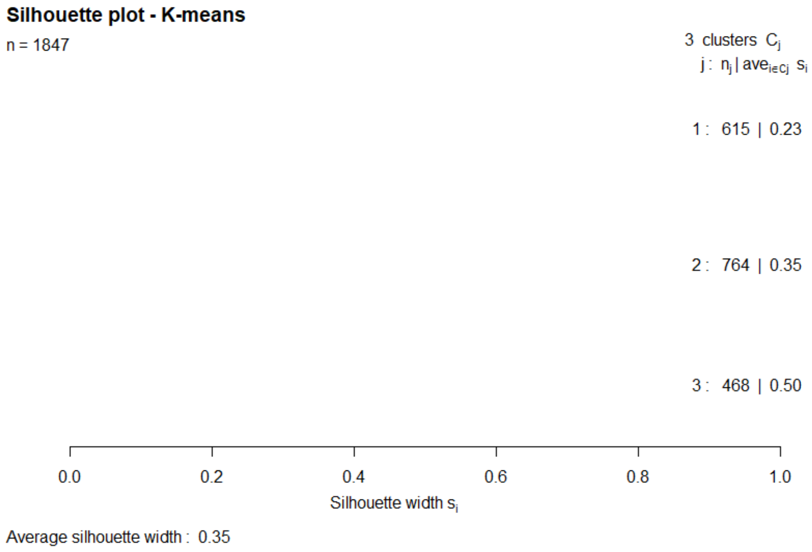

Supplementary data S2

Rutin clustering

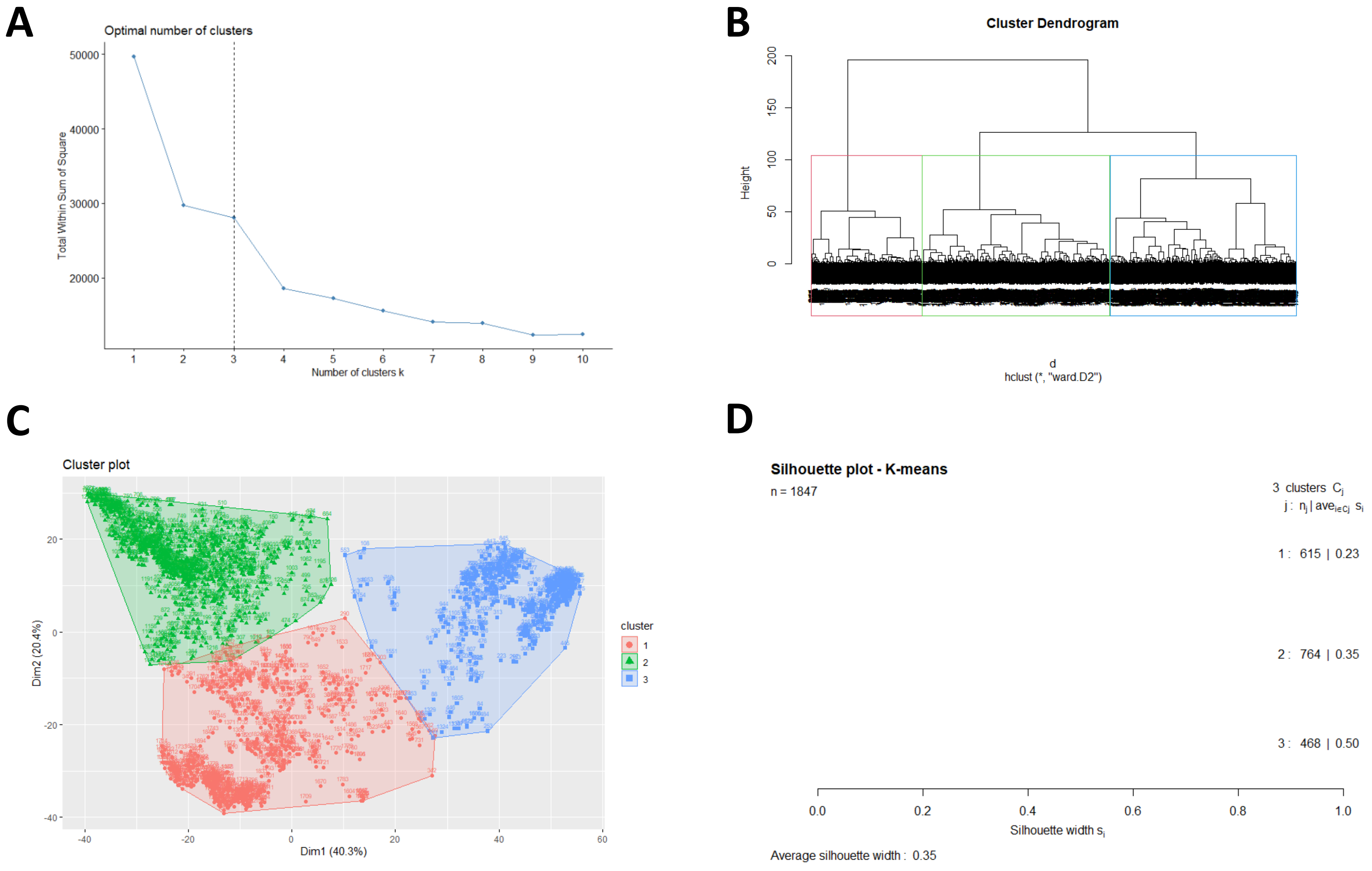

Supplementary data S2

Hidrosmin clustering

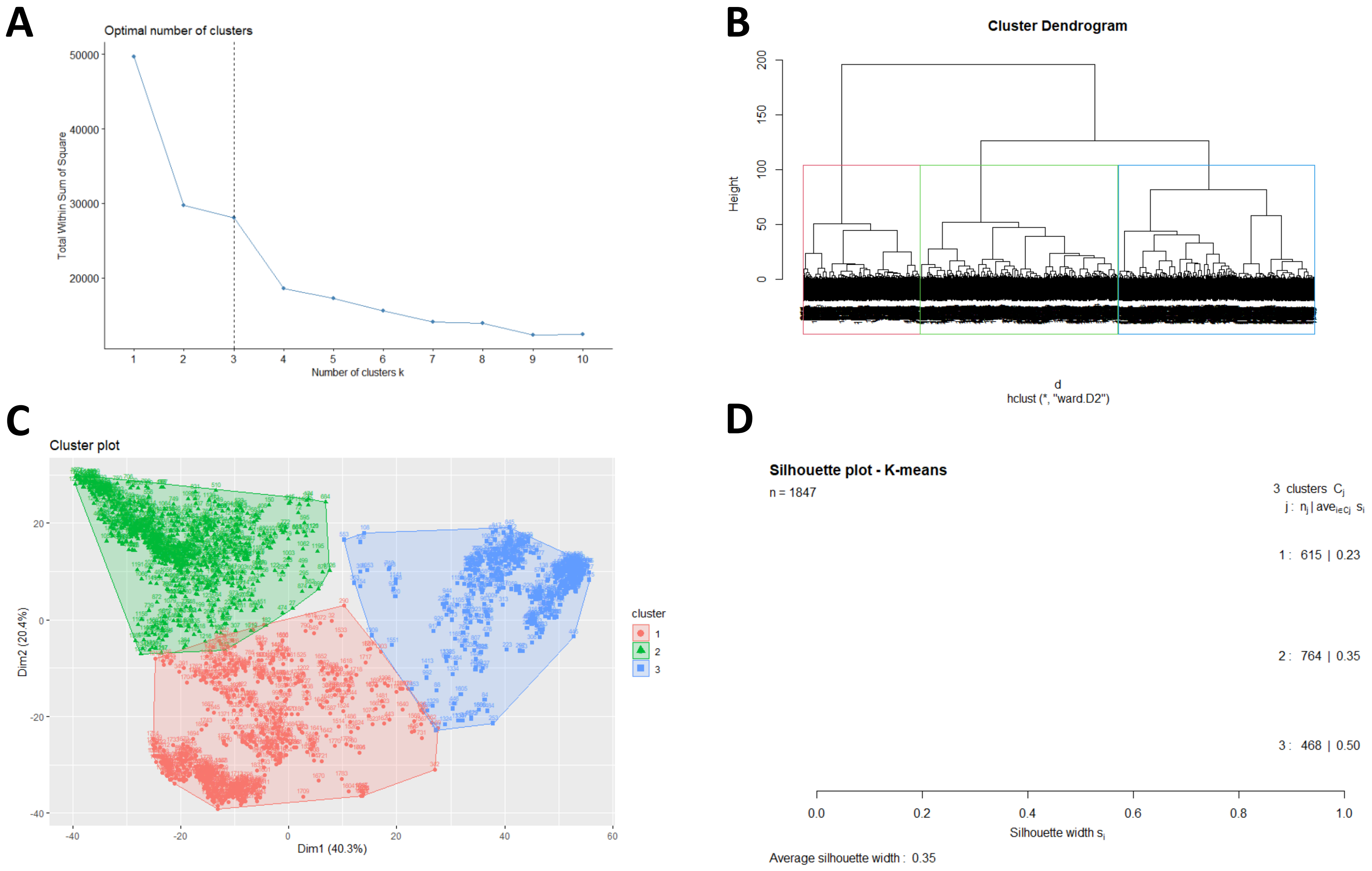

Supplementary data S2

Diosmin clustering

A

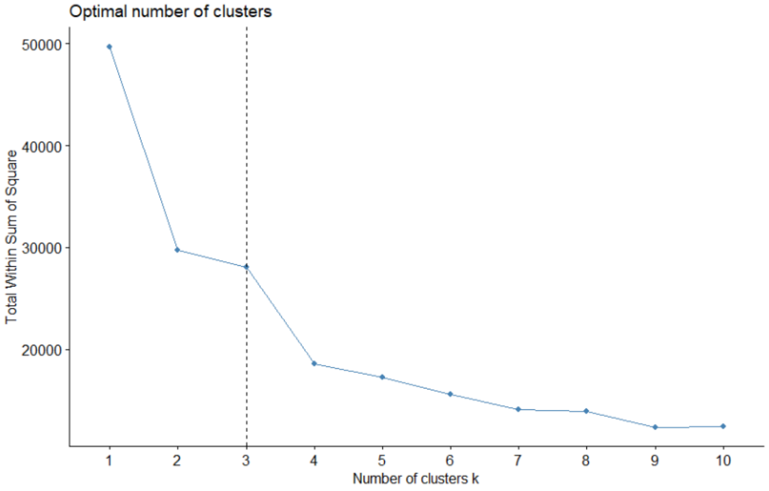

B

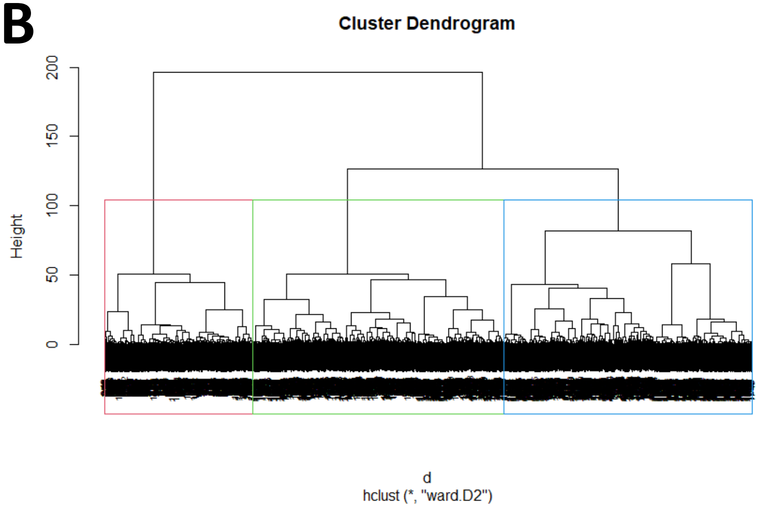

C

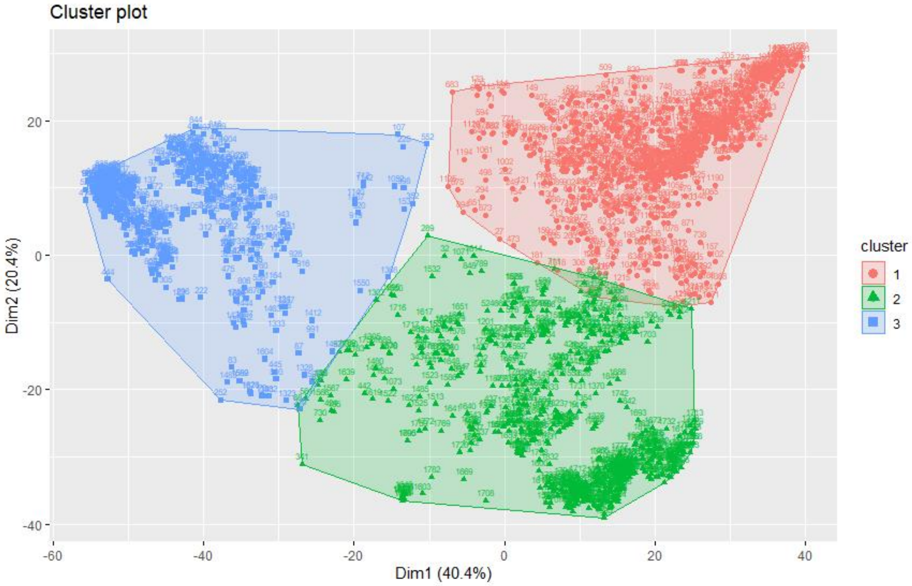

D

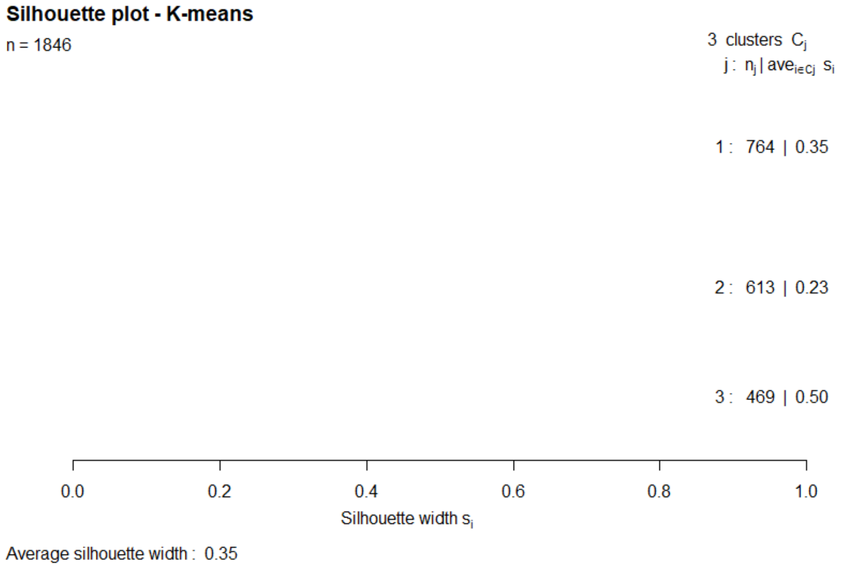

Supplementary data S2

Hesperidin clustering

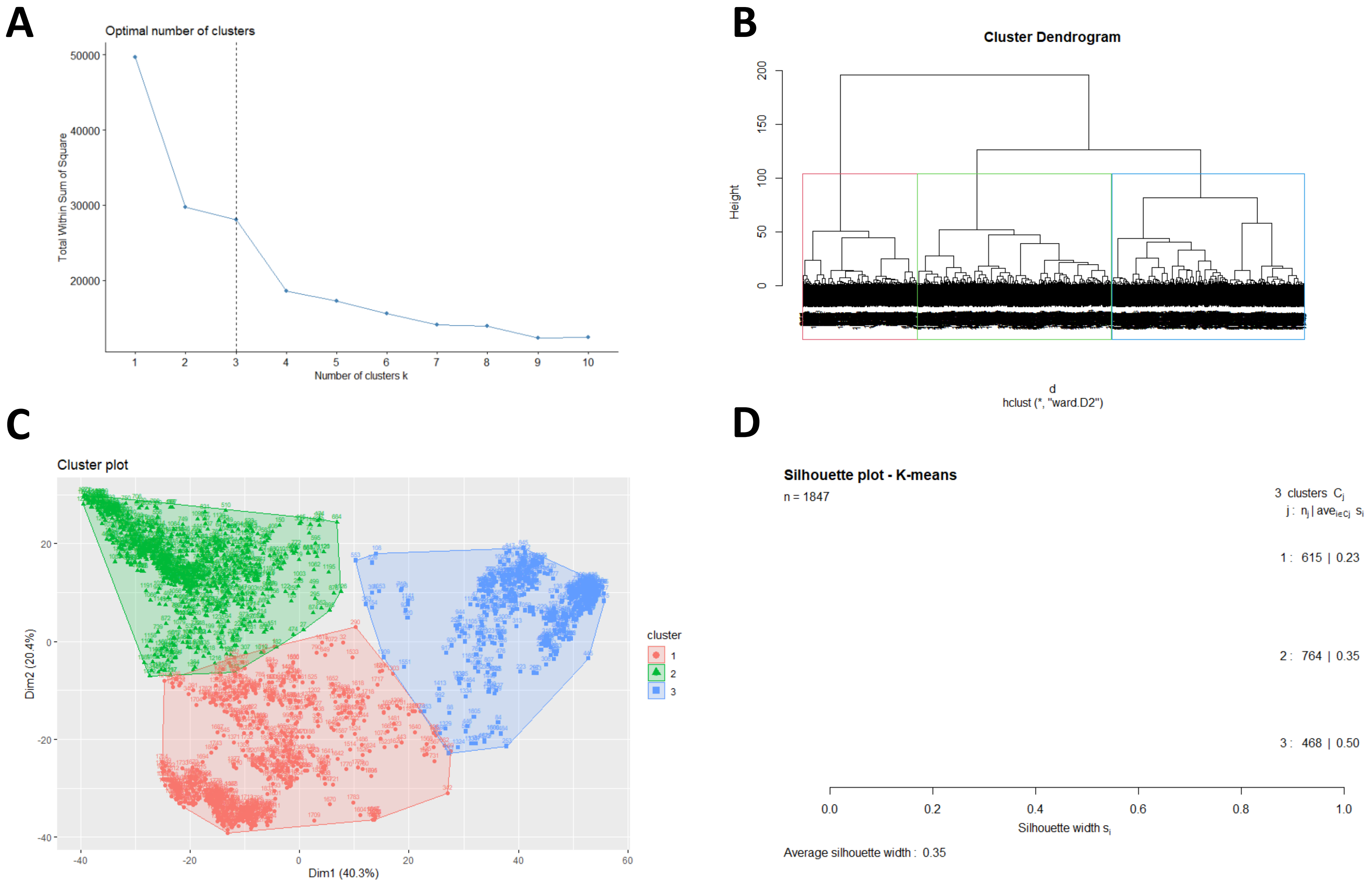

Supplementary data S2

Linarin clustering

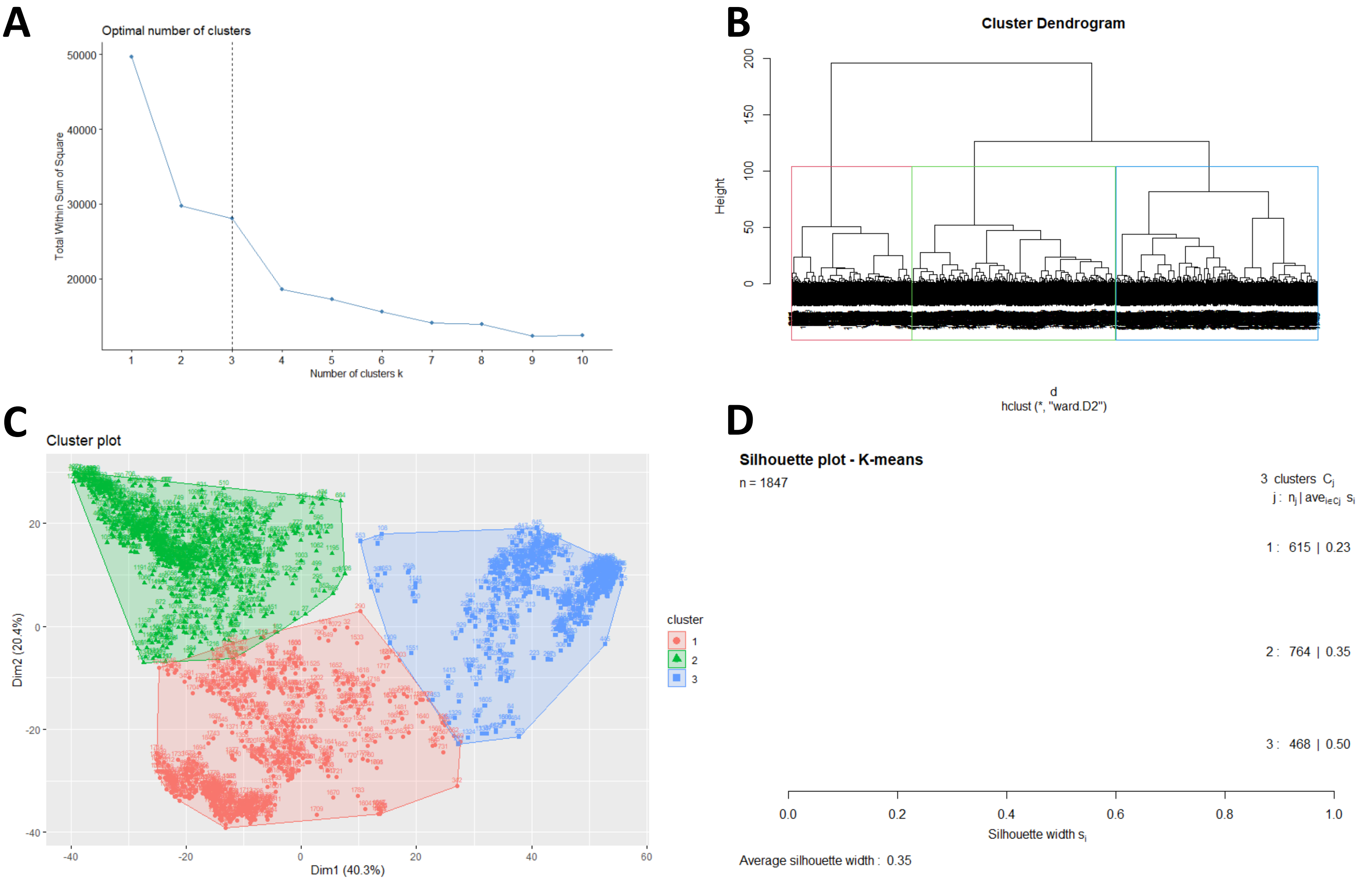

Supplementary data S2

Isorhoifolin clustering

A

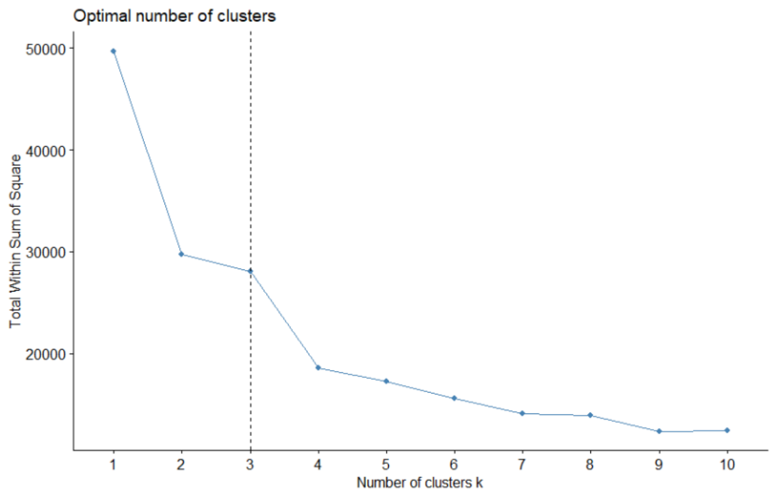

B

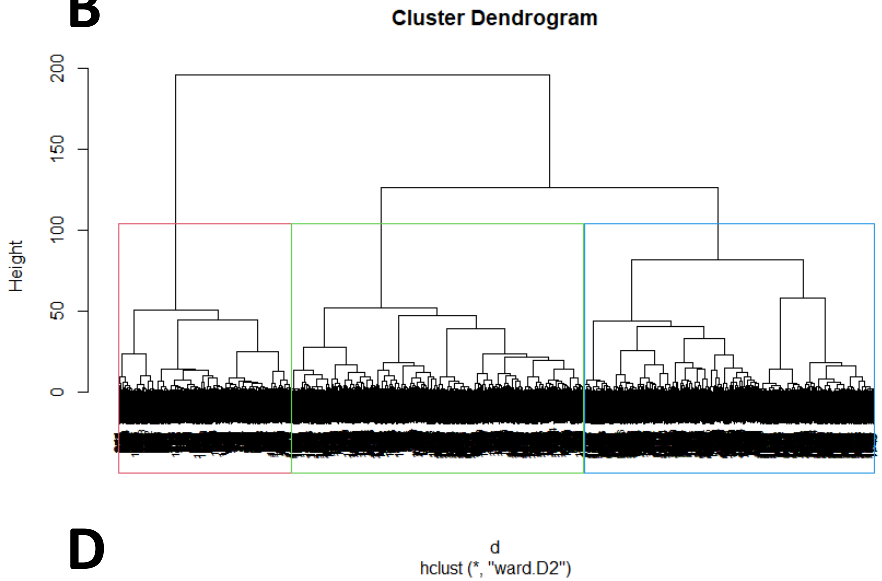

C

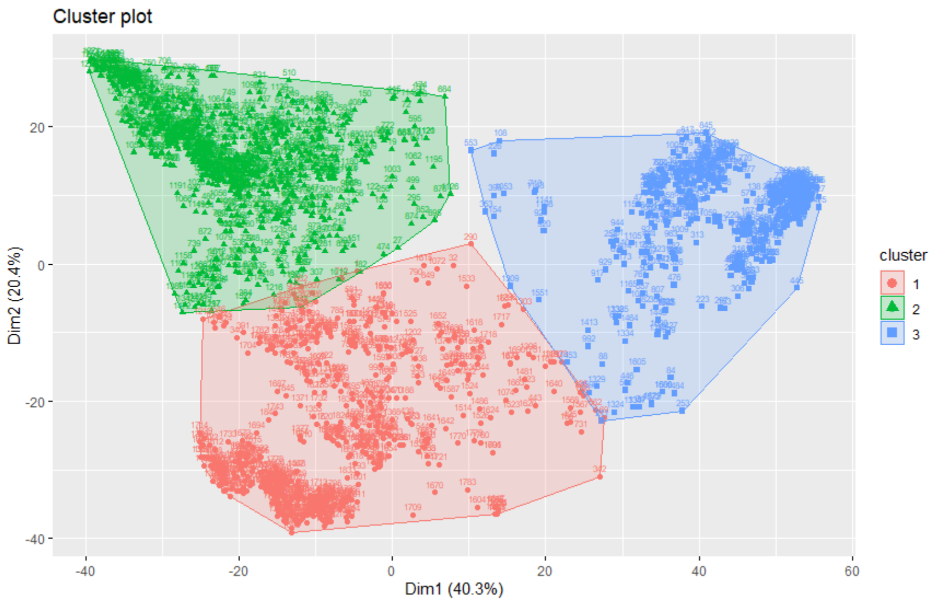

D

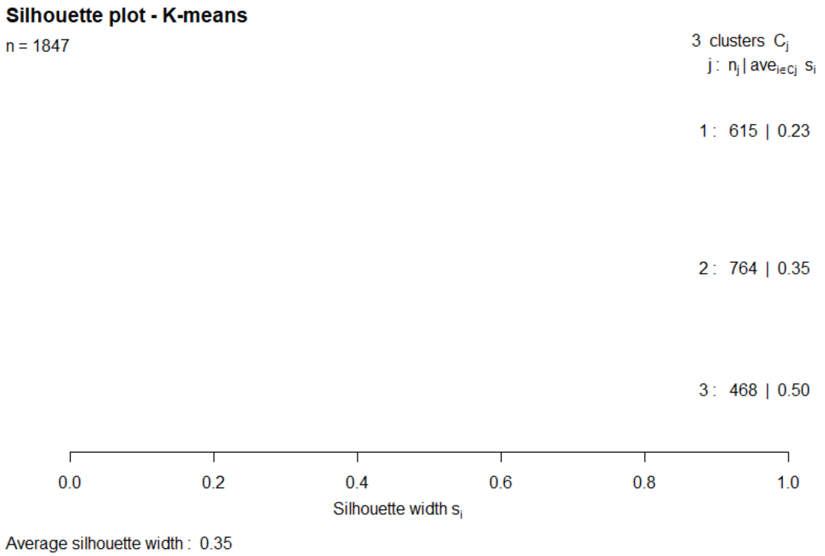

Supplementary data S2

Naringin clustering

A

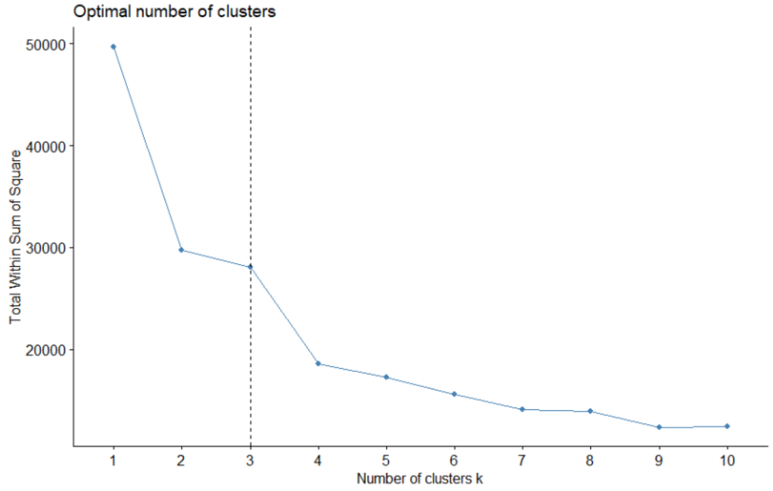

B

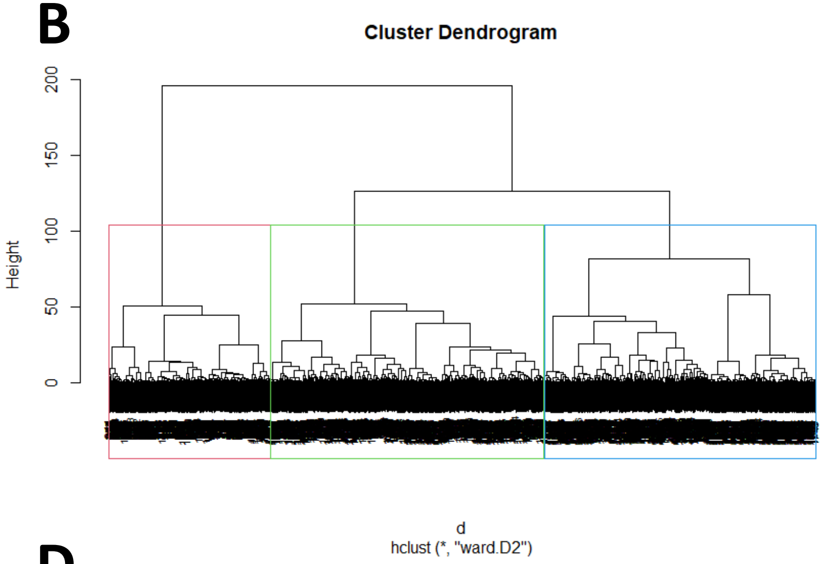

C

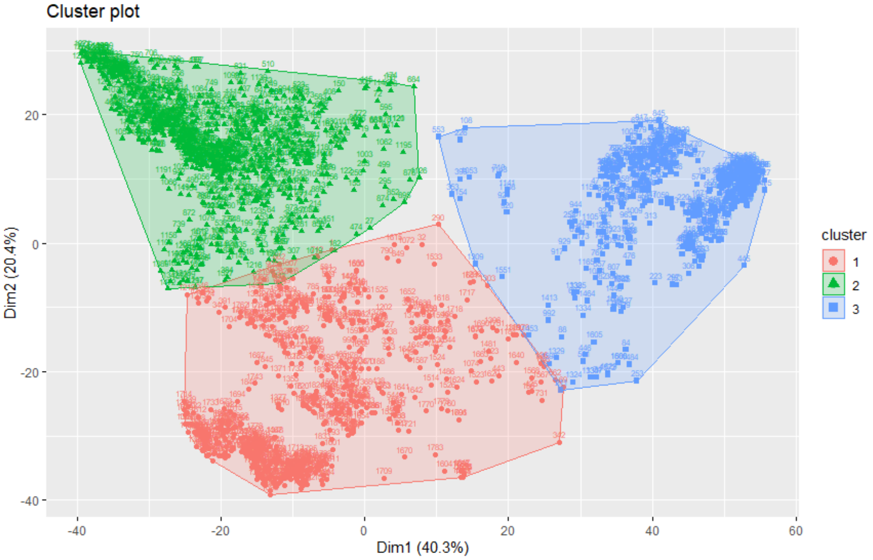

D

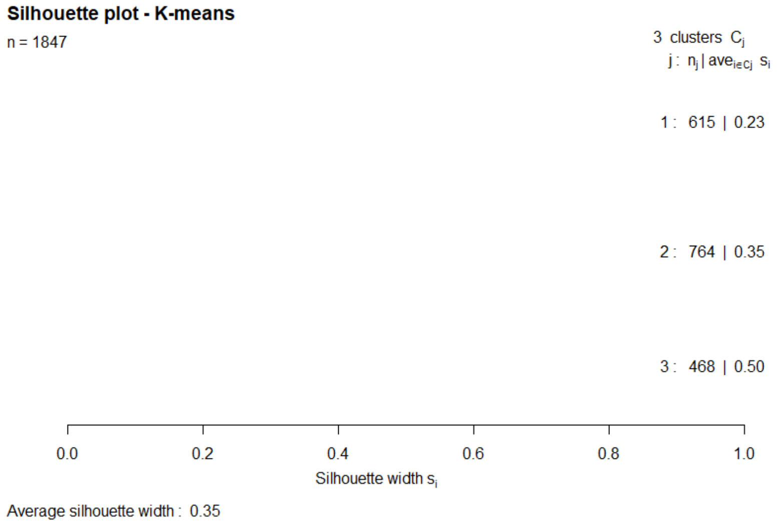

Supplementary data S2

Betanin clustering

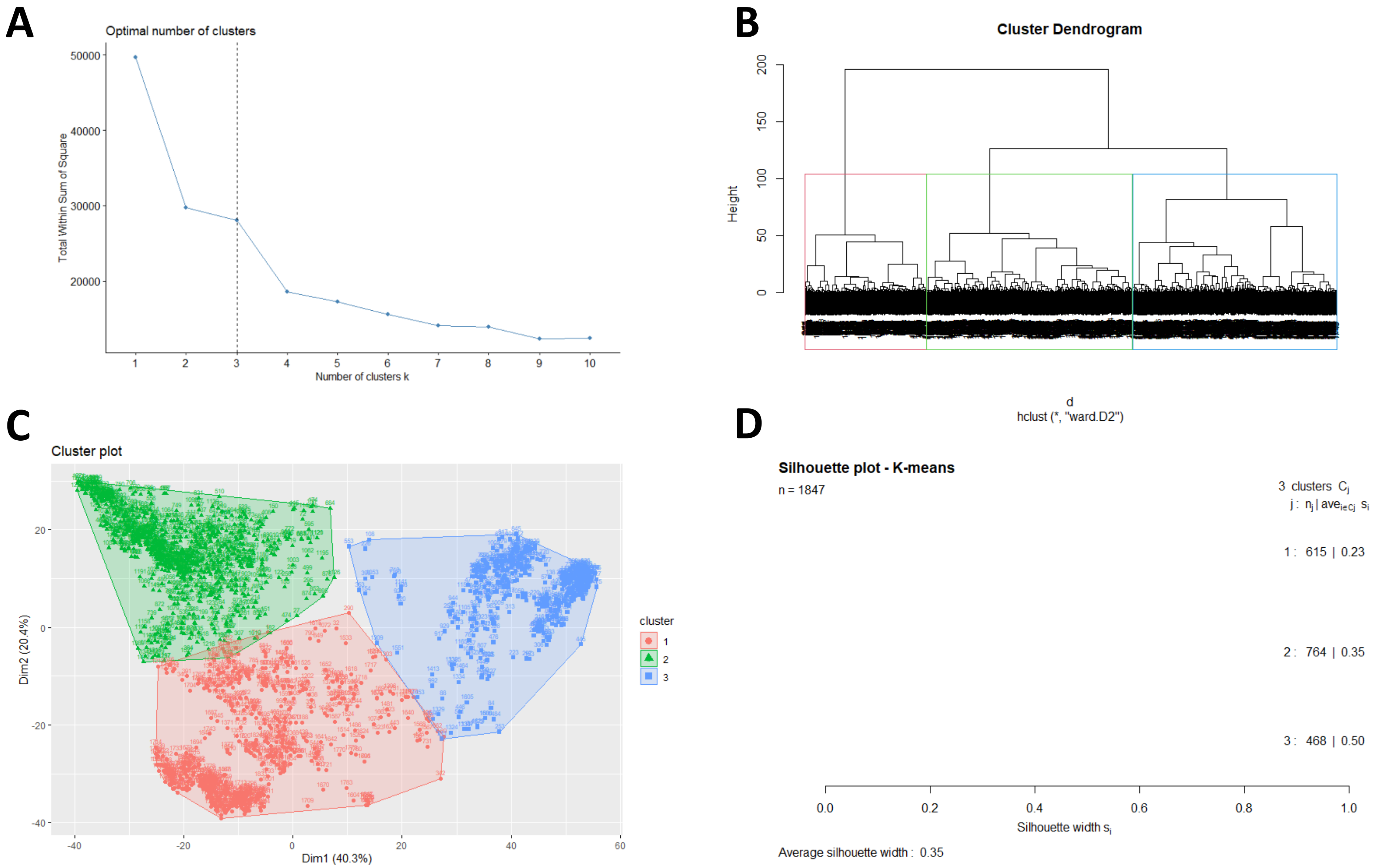

Supplement: Supplementary file 1 [file molecules-28-07937-s001.zip › supplementary_figures.pdf]
